# Non-coding deep learning models for tomato biotic and abiotic stress classification using microscopic images

**DOI:** 10.3389/fpls.2023.1292643

**Published:** 2023-01-08

**Authors:** Manoj Choudhary, Sruthi Sentil, Jeffrey B. Jones, Mathews L. Paret

**Affiliations:** ^1^ North Florida Research and Education Center, University of Florida, Quincy, FL, United States; ^2^ Plant Pathology Department, University of Florida, Gainesville, FL, United States; ^3^ Indian Council of Agricultural Research (ICAR) - National Centre for Integrated Pest Management, New Delhi, India

**Keywords:** diseases, code-free models, machine learning, tomato, deep learning, biotic stress, abiotic stress, microscopic images

## Abstract

Plant disease classification is quite complex and, in most cases, requires trained plant pathologists and sophisticated labs to accurately determine the cause. Our group for the first time used microscopic images (×30) of tomato plant diseases, for which representative plant samples were diagnostically validated to classify disease symptoms using non-coding deep learning platforms (NCDL). The mean F1 scores (SD) of the NCDL platforms were 98.5 (1.6) for Amazon Rekognition Custom Label, 93.9 (2.5) for Clarifai, 91.6 (3.9) for Teachable Machine, 95.0 (1.9) for Google AutoML Vision, and 97.5 (2.7) for Microsoft Azure Custom Vision. The accuracy of the NCDL platform for Amazon Rekognition Custom Label was 99.8% (0.2), for Clarifai 98.7% (0.5), for Teachable Machine 98.3% (0.4), for Google AutoML Vision 98.9% (0.6), and for Apple CreateML 87.3 (4.3). Upon external validation, the model’s accuracy of the tested NCDL platforms dropped no more than 7%. The potential future use for these models includes the development of mobile- and web-based applications for the classification of plant diseases and integration with a disease management advisory system. The NCDL models also have the potential to improve the early triage of symptomatic plant samples into classes that may save time in diagnostic lab sample processing.

## Introduction

Plants contribute up to 80% of food for human consumption and feed for livestock. Numerous pests and diseases cause approximately 20%–40% losses in crop yield, with diseases resulting in $220 billion in losses annually (FAO, 2020). In 2021, tomato was a major vegetable crop grown worldwide with an annual production of 189 million tons from 5.2 million hectares (FAO, 2022). Tomato is affected by almost 200 diseases, and these frequently create bottlenecks for optimum tomato production (Jones et al., 2014). The accurate identification of disease symptoms is the first step in the management of plant diseases. In many cases, incorrect diagnosis of plant diseases leads to mismanagement and economic losses (Riley et al., 2002). Currently, disease identification is done by a combination of visual observations and lab-based techniques (e.g., microscopy, culturing, antibody, and molecular methods). This process can be time-consuming and needs highly trained personnel, who are available in developed countries while limited or not present in developing and less-developed countries. Added to this challenge, some plant diseases can spread like “wildfire” and can destroy a crop in days (Fry et al., 2013; Chen, 2020). Thus, there is a need for faster, cheaper, and reliable disease classification approaches to identify potential causes of disease and selection of management strategies.

Deep learning is a subfield of artificial intelligence (AI). Deep learning refers to the use of artificial neural network architectures that contain a substantial number of internal processing layers to provide processed output from raw data (Lecun et al., 2015). Deep learning tools are used in a wide variety of tasks in agriculture (e.g., disease classification, yield estimation and forecasting, crop genomics, and modeling). In the last decade, several researchers used machine learning-based tools for the classification of plant diseases (Mohanty et al., 2016; Sladojevic et al., 2016; Amara et al., 2017; Brahimi et al., 2017; Ferentinos, 2018; Lee et al., 2020; Liu et al., 2021). For tomato, researchers used various deep learning models like AlexNet, GoogleNet, Inception V3, Residual Network (ResNet) 18, and ResNet 50 (Tran et al., 2019; Maeda-Gutiérrez et al., 2020; Chong et al., 2023); Yolo V3 convolutional neural network (Liu and Wang, 2020); or conditional generative adversarial network (Abbas et al., 2021). Many of these models had long training times (up to 2 weeks) with modest datasets (Picon et al., 2019) and low accuracy with test datasets (Liu et al., 2021). In addition, the models had a lower performance matrix on external field captured images and required resources including 1) high-performance computer with a costly graphic processing unit (GPU) or access to cloud computing and 2) expertise with skills in deep learning.

Researchers working directly with farmers have limited resources and lack robust AI talent pool, especially in developing and underdeveloped countries (Gwagwa et al., 2021). One potential solution to these challenges is to use no coding or minimum coding deep learning models called automated machine learning. This is a process of applying machine learning models to real-world problems by streamlining raw data processing, feature, and model selection and hyperparameter optimization with minimal human inferences (Kanti Karmaker et al., 2021). Recently automated machine learning approaches were tested in the medical field for the classification of images of the eye (Korot et al., 2021), video of cataract (Touma et al., 2022), and carotid injury (Unadkat et al., 2022). Automated machine learning facilitates users to build state-of-the-art models without writing codes, making them accessible to a wide range of individuals without coding experience.

The accurate classification of diseases is extremely challenging as most disease symptoms are randomly distributed within plant parts (Riley et al., 2002). Disease symptoms can vary with cultivar, crop stage, environment, and other biotic and abiotic factors necessitating that the image databases used in building models are updated regularly with new sets of images. Deep learning models already used in plant disease classification have the same image database such as PlantVillage or Kaggle and have led to non-ideal robustness and overfitting, resulting in a reduction in testing accuracy (Mohanty et al., 2016; Darwish et al., 2020). In some cases, classification performance on external data evaluation was reduced below 50% (Mohanty et al., 2016; Ferentinos, 2018). AlexNet and GoogLeNet deep learning model performance was reduced to 31% from 99% with the PlantVillage database (Mohanty et al., 2016). Deep learning VGG model success rate on testing with real field images reduced to 33.3% on laboratory-tested models despite a large dataset of over 80,000 images used for training of the models (Ferentinos, 2018).

Most of the existing database images are taken using a mobile phone or camera. These macroscopic images capture more background features (pixels) beyond the focus on the diseased section of the plant parts. Lab disease diagnosis is based on both visual symptom examination, using a stereoscopic microscope often followed by a molecular diagnostic assay (Riley et al., 2002). Stereoscopic microscopes provide ×25–70 magnification, which helps in better visualization of fungal symptoms and signs like sclerotia, mycelial growth, and bacterial oozing, all of which can help in the diagnosis of plant diseases. Images taken with a higher magnification than normal mobile phone cameras may be a potential solution to improve the accuracy of the models in field conditions as the magnified view of the disease symptoms could help in better training with little or no noise from the background features.

Most deep learning models are data-centric and require a well-curated dataset (Kanti Karmaker et al., 2021). Based on the limitations in the accuracy of models for field disease classification and the potential for using non-coding deep learning (NCDL) platforms to improve a better system of classification, this present study was undertaken. We built a new tomato image database that consisted of field-collected microscopic images using a ×30 mobile-mounted camera lens. These images were trained and tested using NCDL platforms including Amazon Rekognition Custom Label (Custom Label), Teachable Machine from Google (Teachable Machine), Microsoft Custom Azure Vision (Custom Vision), Google AutoML Vision (AutoML), Clarifai, and Apple CreateML (CreateML). The goal of this study is to demonstrate the potential use of microscopic images of plant disease with NCDL platforms for improved classification. The specific objectives were i) to develop a tomato biotic stress microscopic images database and ii) to analyze the potential of non-coding machine learning models for disease classification using microscopic images.

## Materials and methods

### Collection of images and diagnostic validation

The study was designed to collect diverse images from major tomato-growing regions in Florida and Georgia. Images were collected during 2020 and 2021 in 10 different field surveys. The images were collected using a mobile-mounted ×30 camera (GoMicro). The android smartphone Xiaomi Redmi Note 5 Pro 12 MP camera was used for image capture by a single individual (**Supplementary Figure 1**). Images were captured using the GoMicro Detect application in natural sunlight in field conditions.

Images were collected from all symptomatic parts, i.e., leaves (upper and lower sides) and fruits. To reduce background noise and effects from shaking, samples were placed on a flat cardboard surface for image capture. Disease symptoms at different stages were captured to have a broad and diverse image collection. In addition to the images of the symptomatic samples, healthy samples were also collected. When symptoms were too large to be captured in a single microscopic image, multiple images were taken of the symptom region. While capturing the images, the optical zoom of the mobile camera was not used. Representative samples during the surveys were diagnostically identified using microscopic, microbiological, and molecular tools to ensure images were classified correctly (**Supplementary Table 1**). All images were visually examined to remove any that were out-of-focus. Images were labeled based on the diagnostic report provided by the plant disease diagnostic lab at the University of Florida, and expert knowledge of an experienced horticulturist was used when relevant to abiotic issues. A small subset of representative images for each class is presented in **Supplementary Figure 2**.

### Non-coding deep learning platform selection

Six different NCDL platforms were selected based on prior literature and information available through Internet searches. Each platform was reviewed for available features, including access for use in the U.S., cost, type of output, graphical user interface, time taken for analysis, and ease of use by a person with minimal coding experience. A summary of the NCDL platforms and reasons for not using a particular platform is provided in **Supplementary Table 2**. Platforms included in this study were Amazon Rekognition Custom Label, Teachable Machine from Google, Microsoft Custom Vision, Google AutoML Vision, Clarifai, and Apple CreateML. All platforms except CreateML use a Microsoft Windows-based system on the web without downloading locally. CreateML was downloaded in a Mac-based system and run locally using the XCode developer program without active Internet. Teachable Machine and CreateML were freely available without any limitations. For the other platforms, the free tier (the use of the model without making any payment) was used first, and once the free limit was exhausted, the basic tier (cheapest plan) was used with payment.

### Data upload and labeling

All dataset images were labeled according to their diagnostic result/expert knowledge. Each NCDL platform has a distinct way of uploading and storage of the dataset. In the case of the Teachable Machine platform, images had to be uploaded each time for training, and the data were not stored locally. For the other platforms, the uploaded data were stored for further use. Only the AutoML platform allows image upload in CSV format. In all the NCDL platforms except CreateML, data had to be labeled manually after upload. Images were labeled separately as training and testing for the CreateML NCDL platform. A detailed description of each NCDL platform is provided in **Table 1**.

**Table 1:** Non-coding deep learning (NCDL) platform features.

Features	Teachable Machine	Clarifai	Custom Vision	CreateML	Custom Label	AutoML
Needs a user account	No	Yes	Yes	No	Yes	Yes
Data storage on cloud	No	Yes	Yes	Locally	Yes	Yes
CSV format data upload	No	No	No	No	No	Yes
Change in threshold	na	Yes	Yes	na	No	Yes
Trained model download	Yes	No	Yes	na	Yes	Yes
Manual tweaking in parameter	Yes	na	na	Yes	No	No
Parameter can be modified	Epoch, learning rate, batch size	–	na	Iteration, blur, noise, crop, expose, flip, rotate	na	Number of nodes, hosting type
Data upload and time (min)	5–10	10–30	10–30	<5	10–30	30–60
Require prior data classification	No	No	No	Yes	No	No
Time for training	<30 min	<30 min	1–10 h	<10 min	1–20 h	1–10 h
Overall accuracy/F1 score provided	No	Yes	Yes	Yes	Yes	Yes
Confusion matrix	Yes	Yes	No	No	No	Yes
Download trained model	Yes	No	Yes	No	Yes	Yes
Testing on external image with deployment	Yes (individual image)	No	Yes (individual image)	Yes	No	Yes (batch upload)
Testing on external image with deployment	Yes (individual image)	No	Yes (individual image)	Yes	No	Yes (batch upload)
Availability	Free	Free up to a certain level	Free up to a certain level	Free	Free up to a certain level	Free up to a certain level
Cost						
Require credit card information	No	No	Yes		Yes	Yes
Free quota	Na	1,000 operations/month	5,000 images in 2 projects. 1hr of training per month	Na	10 training hours and 4 interface	$300
After free tier		$30/month	$10 per compute hour	Na	Training: $1/h Interface: $4/h	$3.462/node/h for training
Easy to use (1–5)	Easy	Medium	Medium	Medium	Hard	Hard
Data visualization	Good	Excellent	Ok	Ok	Good	Excellent
Model performance	Low	Medium	High	Very low	High	Medium

### Data processing

Training with the supervised NCDL platform needs images to be split into training, validation, and testing sets. All except CreateML did not use prior splitting of the image dataset. Each NCDL platform default setting was used for the splitting of each image dataset, and if manual setting was required, 80:20 (training:testing) split was used. For CreateML, python library split-folders (V 0.5.1) (Split-folders:PyPI, 2022) were used to split the dataset into training and testing datasets of 80:20 ratio. Teachable Machine and AutoML have 85:15 and 90:10 split ratios, respectively. Duplicate images were automatically detected by AutoML and Custom Vision, and these images, if any, were removed from the training databases in all the other NCDL platforms. Custom Vision advanced (no time for training) option was used for training.

### Model training

NCDL platform models were trained using image databases described in **Table 2** using a web interface except in the case of CreateML. These models did not require any specific CPU or GPU uses. CreateML platform was run locally on the Mac-based system that has 256 GB SSD and 8 GB RAM. For the default threshold value of each model, or where a manual option was available, 50% (0.5) was used as the threshold value for classification. The parameters that can be modified within each platform are described in **Table 1**. Unless otherwise specified, we did not use hyperparameters or change the default setting of the NCDL platform models.

**Table 2:** Databases for the training of the non-coding deep learning (NCDL) platform models used in this study.

Database	Symptom class	Number of images	Number of classes
Fruit	Bacterial spot of tomato (BST)	543	
	Healthy	55	
	Pox	121	
	Raincheck	234	
	Tomato spotted wilt (Tospo)	161	
	**Total images**	**1,114**	
Lower side of the leaf	BST	209	
	Early blight	210	
	Healthy	52	
	Little leaf	48	
	Spider mites feeding damage (SMFD)	61	
	Tomato yellow leaf curl (TYLC)	39	
	**Total images**	**619**	
Upper side of the leaf	BST	475	
	Early blight	472	
	Healthy	267	
	Little leaf	125	
	Nutrient deficiency	152	
	SMFD	110	
	TYLC	337	
	2-4-D drift damage	240	
	**Total images**	**2798**	
**Total images**		**3,911**	
Combined individual classes^a^			19
Leaf image combined^b^			13
Leaf and fruit image combined^c^			11

a All individual five classes of fruit, six classes of the lower side of the leaf, and eight classes of the upper side of the leaf used. In this case, none of the image classes were combined.

b Lower side of the leaf class combined with the upper side of the leaf images class to have a single class of images. For example, the BST lower side of leaf images combined with the upper side of leaf images to have BST (leaf) class. All six classes to the lower side of the leaf combined to the respective upper side of the leaf.

c BST and healthy class upper side of the leaf, the lower side of the leaf, and fruit symptom images combined into a single class.

### Metrics and statistical analysis

Each model graphic user interface provides different types of outputs. To compare the models, we used precision/positive predictive value (PPV), negative predictive value (NPV), recall/sensitivity, specificity, accuracy, and F1 score. To evaluate these parameters, we extracted the following: true positive (TP), which is the number of positive images that were classified as the same as the labeled class; true negative (TN), which is the number of negative images that were classified as the same as the labeled class; false positive (FP), which is the number of images classified as positive but do not belong to that labeled class; and false negative (FN), which is the number of images that belong to the labeled class but were classified in a different class. A summary of the approach is given below.


$$PPV/Precision\;\left(\%\right)=\frac{Number\;of\;images\;correctly\;identified\;\left(TP\right)}{Total\;number\;of\;images\;idenfied\;as\;correct\left(TP+FP\right)}\;\times 100$$

$$NPV\;\left(\;negative\;predictive\;value\right)=\frac{TN}{TN+FN}$$

$$Specificity=\frac{TN}{FP+TN}$$

$$Sensitivity/Recall\;\left(\%\right)=\frac{Number\;of\;images\;correctly\;identified\;\left(TP\right)}{Total\;number\;of\;correct\;images\left(TP+FN\right)}\;\times 100$$

$$Accuracy\left(\%\right)=\frac{Correctly\;identified\;positive\;and\;negative\;images\;\left(TP+TN\right)}{Total\;number\;of\;images\;\left(TP+TN+FP+FN\right)}\;\times 100$$

$$F1\;score=\frac{2\;\times Precision\times Recall}{Precision+Recall}$$

Some models provided a confusion matrix, which is an easy way to understand models in terms of each class and how many misclassified images belong to which class in a table format. Graphs were created with precision and recall value, and the area under this graph line referred to as the area under precision–recall curve (AUPRC) was only available in AutoML, mentioned as the average precision.

Each of the NCDL platform model outputs was distinct. The Teachable Machine provides only accuracy and a confusion matrix. Clarifai produces one K spilt value of cross-validation report along with a confusion matrix. Custom Vision provides only precision, recall, and average precision (AUPRC/AP) values. CreateML provides precision and recall for each labeled class and overall accuracy for validation and test data. Custom Label provides the threshold used for the classification of each labeled class and the corresponding precision, recall, and F1 score. The AutoML model provides a confusion matrix and accuracy. The AutoML model precision and recall curve can be modified with an interactive threshold level. A default threshold value of 0.5 was used for all NCDL platforms.

## Results

### Image collection and database

Image databases were created for fruit images (symptomatic and healthy) and for leaf images [symptomatic and healthy adaxial (upper) and abaxial (lower) surfaces]. Overall, a total of 3,911 images were collected and labeled in 19 different classes (**Table 2**). For NCDL platform model analyses, these images were further classified into three different categories: 1) all 19 individual class labeled images were used for analysis and referred as “combined individual class”; 2) individual classes of adaxial and abaxial leaf symptoms were pooled into one class, making 13 total classes, and referred as “leaf images combined”; and 3) finally, images of classes with fruit and abaxial and adiaxial leaf symptoms were pooled in single class making a total of 11 classes and referred as “leaf and fruit images combined.” In total, six different types of image databases were analyzed using different models.

### Deep learning model performance

**Figure 1:**
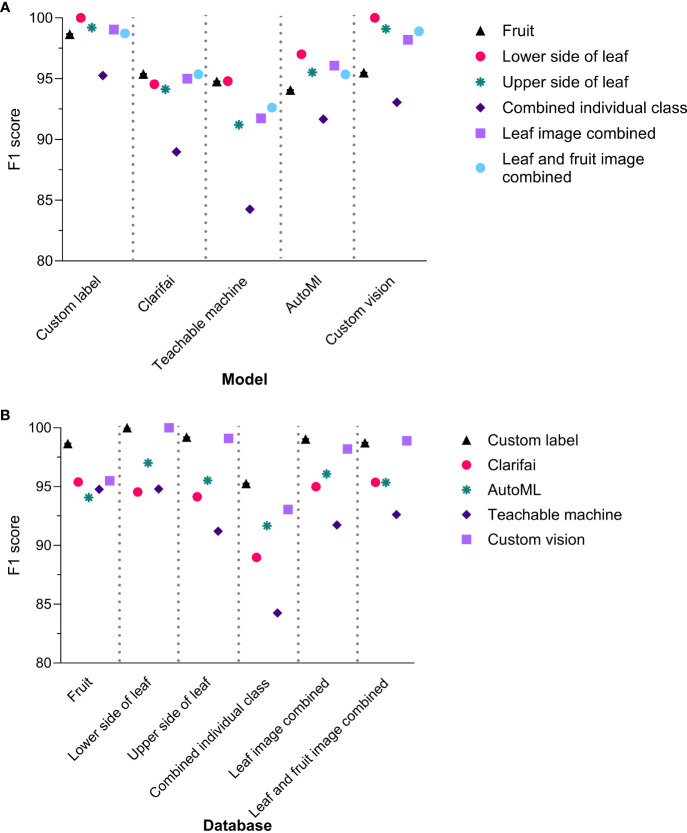
F1 scores of the non-coding deep learning (NCDL) models grouped by **(A)** models and **(B)** database.

The F1 score (Standard Deviation (S.D.)) of the different NCDL platforms across all models-databases were as follows: Custom Label, 98.5 (1.6); Clarifai, 93.9 (2.5); Teachable Machine, 91.6 (3.9); AutoML, 95.0 (1.9); and Custom Vision, 97.5 (2.7) (**Figure 1A** and **Supplementary Table 3**). The F1 score was significantly different between the models (*F*(4, 25) = 6.729, *P* = 0.0008). The Custom Label F1 score was significantly higher than Teachable Machine and Clarifai with Tukey’s multiple *post-hoc* comparisons. The Custom Vision F1 score was significantly different than only Teachable Machine with a mean difference (95% CI) of 5.9 (1.5–10.4) (**Supplementary Table 4**). All the other NCDL models showed statistically similar F1 scores.

Accuracy (S.D.) denotes the percentage of images identified correctly and were as follows: Custom Label, 99.8% (0.2); Clarifai, 98.7% (0.5); Teachable Machine, 98.3% (0.4); AutoML, 98.9% (0.6); and CreateML, 87.3% (4.3) (*F*(4, 25) = 42.29, *P* = 8.412e−11) (**Supplementary Table 5**). CreateML had a significantly lower accuracy among all the NCDL platform models tested with the following mean difference (95% CI): Custom Label 12.4 (9.1–15.7), Clarifai 11.4 (8.0–14.6), Teachable Machine 10.9 (7.7–14.3), and AutoML 11.6 (8.2–14.85) (**Supplementary Table 6**).

Among the different image database–model pairs, F1 scores were as follows: fruit, 95.7 (1.6); lower side of the leaf, 97.3 (2.7); upper side of the leaf, 95.8 (3.4); combined individual class, 90.6 (4.2); leaf images combined, 96.0 (2.9); and leaf and fruit images combined, 96.2 (2.6) (**Figure 1B**). A database-wide comparison resulted in only the lower side of the leaf image showing a higher F1 score than the combined individual class database (*F*(5, 24) = 2.79, *P* = 0.0316) (**Supplementary Table 7**).

### Evaluation based on platform type

CreateML and Custom Vision were not used for all comparisons, as their web-based interface did not provide the number of true positive, false positive, false negative, and true negative that are required for precision, recall, NPV, specificity, accuracy, and F1 value calculations. Clarifai provides individual classes and overall and ROC curve (ROC AUC), but no other model provides a similar output in their interface, therefore was not used for comparison.

### Individual classes

In general, all NCDL platform models had very good disease image classification outputs in all the databases (**Table 3**). The fruit image database performance across five classes was as follows: F1 score (precision, NPV, recall, specificity): Custom Label, 98.7 (98.7%, 99.7%, 98.7%, 99.7%); Clarifai, 95.4 (95.4%, 98.8%, 95.4%, 98.8%); Teachable Machine, 94.8 (94.8%, 98.7%, 94.8%, 98.7%); AutoML, 94.1 (94.1%, 98.5%, 94.1%, 98.5%); and Custom Vision, 95.5 (95.9%, na, 95.1%, na). All NCDL platforms except Clarifai had numerically higher performance with the lower side of the leaf database than the upper side of the leaf database in the respective models. The Clarifai model had a lower accuracy of 98.2% on the lower side of the leaf database compared with 98.5% on the upper side of the leaf database.

**Table 3:** Comparison of the model–database classification performance matrices for non-coding deep learning (NCDL) platforms^a^.

Database	Model	TP	FP	FN	TN	Precision (%)	NPV (%)	Recall(%)	Specificity(%)	Accuracy(%)	F1 score
Fruit											
	Custom Label	222	3	3	897	98.7	99.7	98.7	99.7	99.5	98.7
	Clarifai	207	10	10	858	95.4	98.8	95.4	98.8	98.2	95.4
	Teachable machine	163	9	9	679	94.8	98.7	94.8	98.7	97.9	94.8
	AutoML	111	7	7	465	94.1	98.5	94.1	98.5	97.6	94.1
	CreateML	–	–	–	–	–		–	-	88.0	–
	Custom Vision	–	–	–	–	95.9		95.1	-	98.7	95.5
Lower side of leaf											
	Custom Label	126	0	0	630	100.0	100.0	100.0	100.0	100.0	100.0
	Clarifai	104	6	6	544	94.5	98.9	94.5	98.9	98.2	94.5
	Teachable machine	91	5	5	475	94.8	99.0	94.8	99.0	98.3	94.8
	AutoML	65	2	2	333	97.0	99.4	97.0	99.4	99.0	97.0
	CreateML	–	–	–	–	–		–	-	91.0	–
	Custom Vision	–	–	–	–	100.0		100.0	-	100.0	100.0
Upper side of leaf											
	Custom Label	438	5	2	3059	98.9	99.9	99.5	99.8	99.8	99.2
	Clarifai	385	24	24	2839	94.1	99.2	94.1	99.2	98.5	94.1
	Teachable machine	301	29	29	2281	91.2	98.7	91.2	98.7	97.8	91.2
	AutoML	213	10	10	1551	95.5	99.4	95.5	99.4	98.9	95.5
	CreateML	–	–	–	–	–		–	-	90.0	–
	Custom Vision	–	–	–	–	99.1		99.1	-	99.5	99.1
Combined individual class^b^											
	Custom Label	754	40	35	14162	95.0	99.8	95.6	99.7	99.5	95.3
	Clarifai	638	79	79	12827	89.0	99.4	89.0	99.4	98.8	89.0
	Teachable machine	503	94	94	10652	84.3	99.1	84.3	99.1	98.3	84.3
	AutoML	374	34	34	7310	91.7	99.5	91.7	99.5	99.1	91.7
	CreateML	–	–	–	–	–		–	-	82.0	–
	Custom Vision	-	-	-	-	93.1		93.0	-	93.8	93.0
Leaf image combined^c^											
	Custom Label	781	8	7	9448	99.0	99.9	99.1	99.9	99.9	99.0
	Clarifai	721	38	38	9070	95.0	99.6	95.0	99.6	99.2	95.0
	Teachable machine	544	49	49	7106	91.7	99.3	91.7	99.3	98.7	91.7
	AutoML	392	16	16	4880	96.1	99.7	96.1	99.7	99.4	96.1
	CreateML	–	–	–	–	–		–	-	91.0	–
	Custom Vision	–	–	–	–	98.2		98.2	-	98.4	98.2
Leaf and fruit image combined^d^											
	Custom Label	779	11	9	7869	98.6	99.9	98.9	99.9	99.8	98.7
	Clarifai	700	34	34	7306	95.4	99.5	95.4	99.5	99.2	95.4
	Teachable machine	540	43	43	5787	92.6	99.3	92.6	99.3	98.7	92.6
	AutoML	389	19	19	4061	95.3	99.5	95.3	99.5	99.2	95.3
	CreateML	–	–	–	–	–		–	-	91.0	–
	Custom Vision	–	–	–	–	98.9		98.9	-	91.0	98.9

a TP, true positive; FP, false positive; FN, false negative; TN, true negative; NPV, negative predictive value. TP,FP,TN and FN were calculated for symptom each class (refer **Table 2**) and pooled data were presented.

b All individual five classes of fruit, six classes of the lower side of the leaf, and eight classes of upper side of the leaf used. No image class was combined.

c Lower side of the leaf class combined with the upper side of the leaf images class to have a single class of images. For example, BST lower side of leaf images combined with the upper side of leaf images to have BST (leaf) class. All six classes to the lower side of the leaf combined to the respective upper side of the leaf. Symptom class wise details were provided in **Supplementary Table 6**.

d BST and healthy class upper side of the leaf, lower side of the leaf, and fruit symptom images combined into a single class.

### Combined classes

The NCDL platform models trained on a diverse combined individual class database had relatively lower model-all classes classification performance with F1 score (precision, NPV, recall, specificity) as follows: Custom Label, 95.3 (95.0%, 99.8%, 95.6%, 99.7%); Clarifai, 89.0 (89.0%, 99.4%, 89.0%, 99.4%); Teachable Machine, 84.3 (84.3%, 99.1%, 84.3%, 99.1%); AutoML, 91.7 (91.7%, 99.5%, 91.7%, 99.4%); and Custom Vision, 93.0 (93.1%, na, 93.0%, na) (**Table 3**, **Supplementary Table 8**). For example, for bacterial spot (BST), out of 91 test set images from the upper side of the leaf, 73 images were classified correctly, while 16 images were classified as false negative for the lower side of BST symptoms, and two false negatives were identified as early blight symptom in the Clarifai model (**Supplementary Table 9**).

Classification matrices improved when the upper and lower sides of the leaf images were combined into one class comparison to all combined individual classes database (**Table 3**). The Custom Label F1 score increased from 95.3 in the combined individual class to 99.0. The F1 score of the lowest performing model, the Teachable Machine, increased from 84.3 to 91.7. In most of the individual classes, the F1 score was above 90, except for the fruit pox symptom (**Supplementary Table 8**). Pox had an F1 score of 90.6, 70.6, 78.9, 76.2, 69.5, and 79.2 with Custom Label, Clarifai, Teachable Machine, AutoML, CreateML, and Custom Vision, respectively (**Supplementary Table 8**). Other classes with low F1 score in CreateML were little leaf (84.7), BST on leaf (83.4), and 2-4-D (2,4-dichlorophenoxyacetic acid) drift (88.8) symptoms.

**Figure 2:**
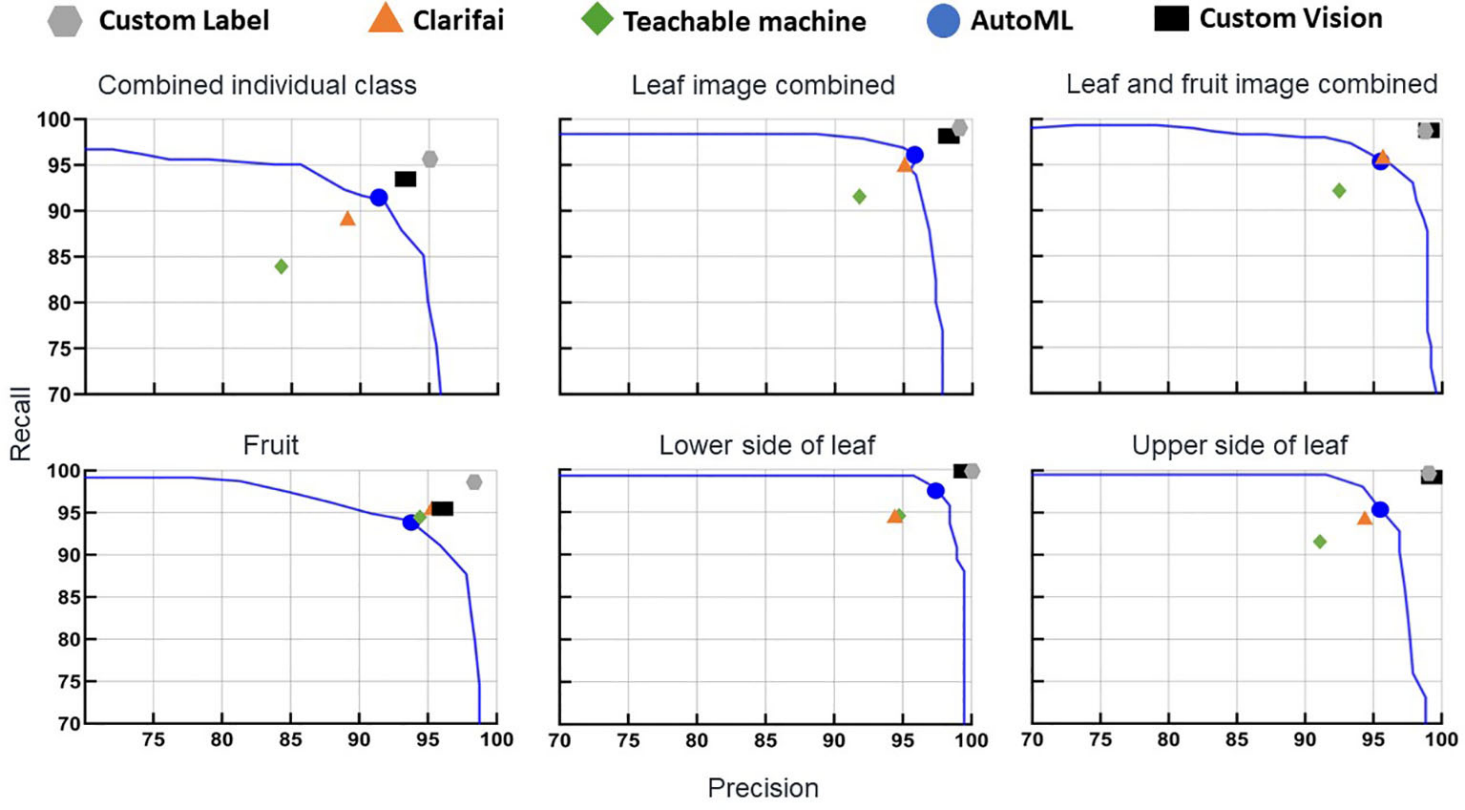
Precision and recall of the non-coding deep learning (NCDL) platform models, with plots grouped by image database. Each point is an individual model’s precision and recall at the default threshold, plotted against the AutoML platform precision–recall curves.

To explore further, symptoms of the same disease on fruit and leaf symptom image classes were combined into one class (BST and healthy class fruit and leaf images combined). Overall, this image database had no impact on either accuracy or F1 score (**Figures 1**, **2**). The F1 score of the leaf and fruit image combined database of Custom Label (99.0 to 98.7) and AutoML (96.1 to 95.3) dropped slightly, while Clarifai (95.0 to 95.4), Teachable Machine (91.7 to 92.6), and Custom Vision (98.2 to 98.9) increased slightly compared with the leaf images combined database.

### External validation of data

**Figure 3:**
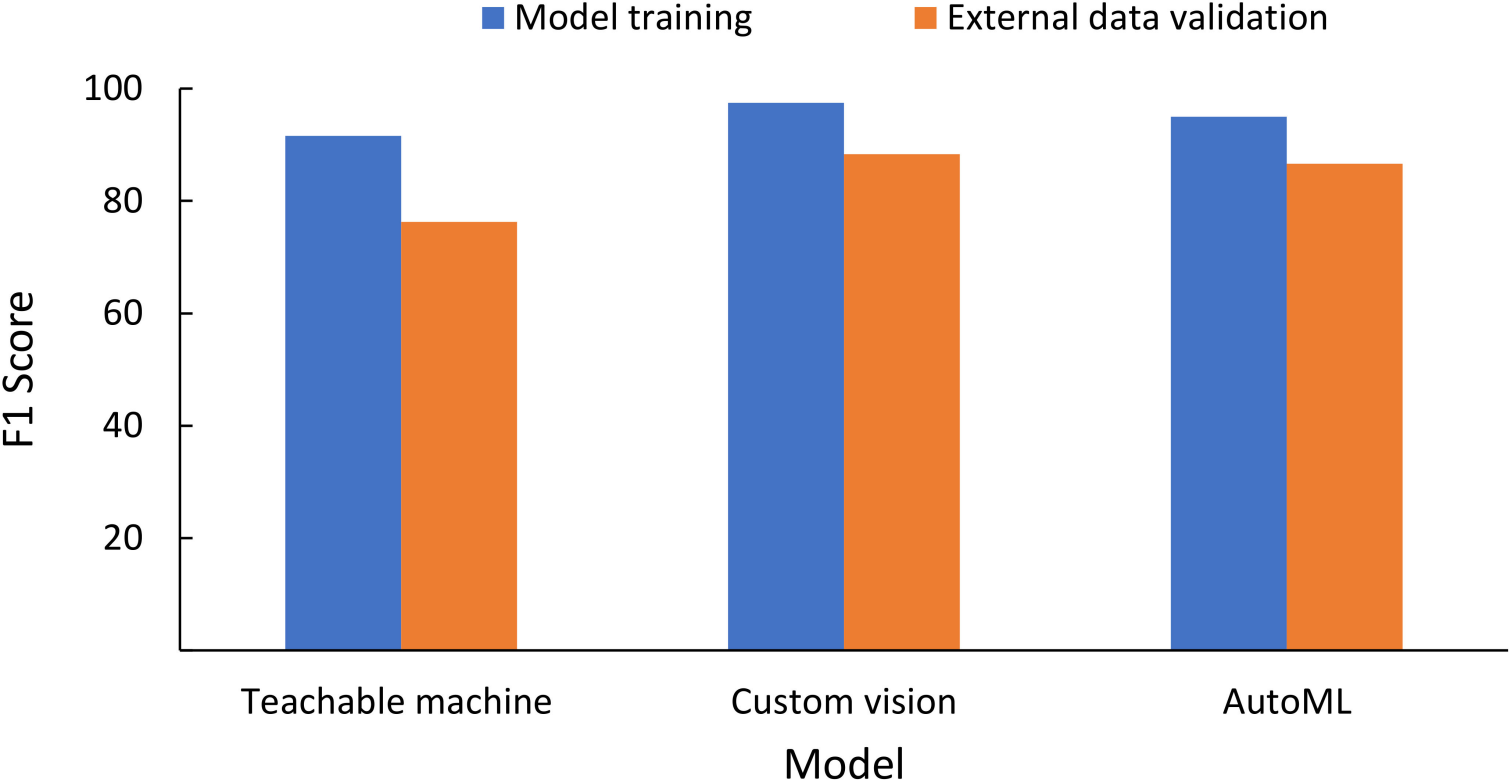
Testing classification matrices of the non-coding deep learning (NCDL) platform models on the external image database (image collected by an independent user and not used in training, testing and validation of the models) using the leaf image combined database model.

External validation of the data was conducted for images collected using ×30 microscopic images by another independent user (Sentil et al., unpublished) using an iOS-based mobile phone on the leaf image combined database models for three NCDLs (i.e., Teachable Machine, Custom Vision, and AutoML). Clarifai and Custom Label do not have an option of testing without deployment of the model and, therefore, were not used. Teachable Machine and Custom Vision only allow testing of individual images, while batch prediction was done with the AutoML model. From a random selection of 200 images, classification performance F1 scores (precision, NPV, recall, specificity) were as follows: Teachable Machine, 76.3 (76.3%, 95.3%, 76.3%, 95.3%); AutoML, 86.6 (86.6%, 97.3%, 86.6%, 97.3%); and Custom Vision, 88.3 (88.3%, 97.7%, 88.3%, 99.7%) (**Figure 3**, **Supplementary Table 10**). The overall accuracy of CreateML in testing data with 50 iterations was 69% (data not shown).

### Repeatability of model

The leaf image combined database was trained on five models three times. The F1 scores (SD) of the models were 89.9 (0.2) for Custom Label, 95.2 (0.4) for Clarifai, 91.4 (3.6) for Teachable Machine, 96.2 (1.2) for AutoML, and 98.6 (0.6) for Custom Vision. The standard deviation was low except for the Teachable Machine, indicating the repeatability of model classification performance.

### Model features

The use of NCDL platforms for plant disease classification is a relatively new area, so each model was examined for various user features from a plant pathologist’s point of view (**Table 1**). Each model has its own set of advantages and technical specifications that need to be worked out. Teachable Machine has the simplest graphic user inference that allows three changeable parameter epochs, batch size, and learning rate. Teachable Machine is the only model in which data need to be uploaded from a local computer or Google Drive, each time the model is trained. The Teachable Machine and CreateML platforms do not require a user account. CreateML models run on a local computer and allow image augmentation like blur, focus, flip, and rotate, which none of the other models have. Modification of the classification threshold allows the user to train models for higher and lower confidence with each classification. Clarifai, Custom Vision, and AutoML allow tweaking of the threshold values. In Custom Label, each class had a different threshold for disease classification at a system-chosen threshold, but this cannot be selected by the user. After uploading, images need to be manually class-wise-labeled in all models. Only AutoML allows data upload in CSV format which makes the process faster than the other models. All NCDL platforms except CreateML automatically separate the testing and training datasets. CreateML requires preclassified testing and training datasets.

CreateML and Teachable Machine were free without any restrictions in use. Clarifai allows 1,000 operations per month in the community tier; after this, monthly charges are required. There were no mentioned criteria for operation, but in this study, we did not run out of free tier quota. Custom Vision has a free limit of 5,000 training images per project and a maximum of 2 projects with a speed of 2 transactions per second (TPS). After free usage, it charges computing hours. The Custom Label free tier allows 10 training hours and 4 interface hours after, hourly training, and interface charges. Custom Label also provides up to 5 GB of image storage in the free tier. AutoML provides initial credit in the free tier, and it charges per node per hour basis for training. Each model has other associated costs for deployment and prediction, which were not used in this study. The use of all the NCDL platform models comprised of three steps: upload and labeling of data, training, and output visualization. Based upon these parameters, Custom Label, AutoML, and Custom Vision models look promising for future uses.

## Discussion

There are many models already available in the web domain, but issues persist with their practical use and field-based validation. Siddiqua et al. (2022) evaluated 17 different mobile applications for disease classification and noted that only “Plantix–your crop doctor” has the capability of disease identification to recommend a suitable management option to users (CGIAR, 2017, https://plantix.net/en/). Some of these models are available for specific crops like “Cassava Plant Disease Identify” for cassava disease and “Tumaini” for banana disease (Selvaraj et al., 2019). However, these mobile application models cannot be customized by local pathologists or extension agents on a local database of choice to improve disease classification (Siddiqua et al., 2022). Microscopic images used in this study have a magnified view of disease symptoms that captures minute characteristics in symptoms than images taken by a normal camera (macroscopic images). Most macroscopic images have the noise of background features in addition to the disease symptoms that make feature extraction more complicated and, thus, reduce accuracy (Lu et al., 2021; Tugrul et al., 2022).

In this study, we evaluated models and multiple combinations of databases using the same number of identical training images using freely available resources in the NCDL platform. Most models (Custom Label, Custom Vision, and AutoML) have similar higher classification performance. Teachable Machine was the least performing model among all web-based cloud computing models tested. If we include locally run models also, then CreateML had significantly lower classification accuracy among all the tested models. Similar findings were also noted when using ophthalmology images with multimodality databases on the CreateML platform (Korot et al., 2021). The exact reason why these two models have poorer performance compared with others for disease classification is unknown as most platforms neither provide information on their configuration and nor allow users to change parameters. Teachable Machine which was built using *Tensorflow.js* works mostly on transfer learning approaches (Teachable machine, 2022) and may have computational limits or may exclude some image data. We used different hyperparameters like epoch and learning rate for Teachable Machine and number of iterations, blur, and focus for CreateML to improve the performance, but it was not significantly improved compared with the default setting (data now shown). As both models (Teachable Machine and CreateML) were freely available without any limit, it is possible that the developers were not routinely improve these models.

The average F1 score of NCDL models ranged from 91.6 to 98.5, while accuracy ranged from 87.3% to 99.8%. Earlier researchers used mostly macroscopic image databases from the PlantVillage database for plant disease classification using the CNN model. Most of the earlier models (AlexNet, GoogLeNet, ResNet, MobiNet, R-CNN, D-CNN) were trained on the same set of images with different augmentations from scratch, or the transfer learning approach has classification matrices from 76% to 99.8% (Abbas et al., 2021; Tugrul et al., 2022). Very few models were tested using external images, and the ones that were tested had a disease classification performance of below 50% (Mohanty et al., 2016; Ferentinos, 2018). Performance reduction on external validation is a major obstacle to the practical application of deep learning models. In our study, only five NCDL models were capable of external validation of the model, in which the F1 score of AutoML and Custom Vision was greater than 86, while Teachable Machine had an F1 score of 76 on a leaf image combined dataset. For the NCDL models, accuracy was reduced by a maximum of 7% in the case of Teachable Machine (the lowest performing model in this study) in external data testing. This indicates that the NCDL platform models were not overfitting or leaking data during the training of the models. In the medical field, models used with eye images have a similar F1 score of as high as 93.9 to the Custom Label models (Korot et al., 2021).

Most leaf and fruit databases had similar classification performance. Only the lower side of the leaf had statistically higher classification performance than the “all individual class” database. This may be due to two reasons: 1) when individual classes were combined, the same type of symptom from the upper and lower side of the leaf images had more false positives and negatives than other databases. The bacterial spot of tomato (BST) upper side of the leaf had 18% (16 out 91) false-positive images as lower side of the leaf. This may be due to early disease symptoms being observed first on the lower side of the leaf and expanding to the upper side of the leaf (Osdaghi et al., 2021). 2) It has also been observed that the lower side of the leaf has more characteristic early and uniform symptoms than other plant parts (Schor et al., 2016). In our study, tomato fruit pox, a genetic disorder (Crill et al., 1973) that produces an incipient oval-shaped lesion, had the lowest classification performance in all databases. Pox was misclassified with fruit BST symptoms and in some cases of healthy samples. This exemplifies issues with the background of images that can reduce classification accuracy. This type of Image-based classification can be further improved using a convex lens that can take the curvature of the plant stem and fruit into consideration and reduce the blurriness of the image.

In terms of the evaluation performance, the top three models (Custom Label, AutoML, and Custom Vision) had no significant differences. Most of the NCDL platforms took less than 1 h for the training of the models. CreateML was the fastest, taking<5 min to create the models. Other models (Custom Label, AutoML, and Custom Vision) took approximately 10 h with the largest dataset. Most models are freely available; however, these models are free only up to certain levels which can be quickly drained. In our analysis, we ran out of free usage in all models except Clarifai. The cost associated with these models is not cheap: Custom Vision charges as high as $10/h/node, which is comparatively costly for a developing nation model developer. Although cloud-based NCDL platforms are easy to use and infinity-scalable, plant pathologists must think about cost requirements compared with the already available traditional methods or outsourcing using machine experts with local resources.

### Limitations with microscopic image collection and models

Even though image collection is quite simple with a microscopic lens, there are no public databases available as of now, and it will take time to build a database of the nature used in this study. A database of such nature that is being built at the University of Florida for tomato, cucurbits, and pepper diseases currently has >30,000 images and is expected to be available for public use by next year (Paret, personal communication). Quality images are necessary for the successful implementation of any AI model. In a recent survey, 96% of the respondents (scientists, AI experts) faced challenges with quality data for the training of ML algorithms (Research Dimensional, 2019). Given that microscopic images provide a magnified view, some diseases like early blight of tomato symptoms may not be captured in a single image, which would increase the number of images to be taken of each class. In diseases like tomato *Fusarium* wilt, which shows dropping of the leaves and yellowing symptoms, whole-plant images in addition to microscopic images may be necessary. In the ideal scenario, a combined approach would be to use both magnified and whole-plant images. However, the machine learning process toward integrating macro- and microscopic images first needs to be established. Another aspect for consideration is the different shapes of the fruits that make it difficult to capture images. The convex shape of tomato fruit may lead to distorted and blurred images. A flexible outing cover for the lens reduces blur due to the shape of the leaf and fruit and facilitates easy adjustment of the camera/lens.

Threshold is an important parameter to have classification with more confidence (Ferri et al., 2019), but the high-performing model such as —Custom Label—did allow modifying this value. Most models do not have an option to change any hyperparameter that makes these models opaque. We do not have information on what kind of parameters and underlying architecture was used by NCDL platform models that reduce the option for improvement in classification performance (Liang et al., 2022). In external evaluation, cloud-based models were lagging a lot. Custom Label and Clarifai do not have the option for external validation before deployment. Teachable Machine and Custom Vision allow individual images for testing that make testing cumbersome and time-consuming. AutoML allows batch processing, but preparing data again requires a laborious process.

### Potential of microscope image coupled NCDL platform

Globally, 95% of the population has mobile network coverage that makes mobile use accessible everywhere (ITU, 2021). In 2021, 5.34 billion people had smartphones which increased by 73% within 5 years (2016–2021) (DataReportal, 2022). This study demonstrates the potential use of mobile phones integrated with deep learning models for plant disease classification. Currently, a major challenge in agriculture in remote places of the world is real-time advisory to endpoint stakeholders. Various down-term applications can be easily integrated with the NCDL platform. Trained models can be utilized as a research tool, deployed on new databases or on prospectively curated data, to collate images that fit a criterion. These potential use cases are not comprehensive, and more will be revealed as agriculture researchers gain an understanding of ML principles through the exploration of NCDL platforms.

AI progression is similar to what has occurred in other technologies (sequencing, mobile, etc.) first by a small core group of scientists, later by experts that navigate the technical nuance of new development, and finally by the general public. Slowly, AI is coming to the domain of “citizen science” where it can be used to reap benefits (Craig, 2022). We explored only a limited number of publicly available NCDL platforms. In coming times, advancement in NCDL platforms and more use of these platforms likely will persuade other big AI companies to improve these models. For now, though, most no-code AI users are business professionals who want to streamline the way things are done without having to involve a programmer.

## Conclusion

We evaluated the classification matrices and usability of six NCDL platforms using microscopic-magnified ×30 images captured using a mobile phone camera-mounted lens. The outcomes from the study demonstrate that the NCDL platform-developed models improved disease classification. Our finding illustrated that plant disease classification using microscopic-magnified images in combination with NCDL models can be used by any plant pathologist and diagnostician for the recommendation of disease management measures to farmers. This study demonstrates the potential use of mobile phones integrated with deep learning models in remote corners of the world without the help of computer personnel. The methods used in this work can speed up disease identification and save a lot of resources (chemicals, lab space, and personnel). The NCDL platforms have a broad scope for agriculture, in which it can potentially be used for understanding nutrient limitations, weed identification, and insect diagnosis. Currently, a major challenge in agriculture in remote places of the world is real-time advisory to endpoint stakeholders. This technology should improve real-time advisory capabilities in remote places.

## Data availability statement

The datasets presented in this study can be found in online repositories. The names of the repository/repositories and accession number(s) can be found below: https://github.com/manoj044/Tomato_microscopic_images.git.

## Author contributions

MC: Conceptualization, Data curation, Formal analysis, Investigation, Methodology, Software, Writing – original draft, Writing – review & editing. SS: Investigation, Writing – review & editing. JJ: Conceptualization, Funding acquisition, Methodology, Supervision, Writing – review & editing, Project administration. MP: Conceptualization, Funding acquisition, Investigation, Methodology, Supervision, Validation, Writing – review & editing.
