# Supplementary material for: Non-coding deep learning models for tomato biotic and abiotic stress classification using microscopic images
Source: Front Plant Sci. 2023 Jan 8;14:1292643. doi: 10.3389/fpls.2023.1292643 (PMC10800394; doi:10.3389/fpls.2023.1292643)
Supplement: Supplementary file 1 [file Image_1.pdf]

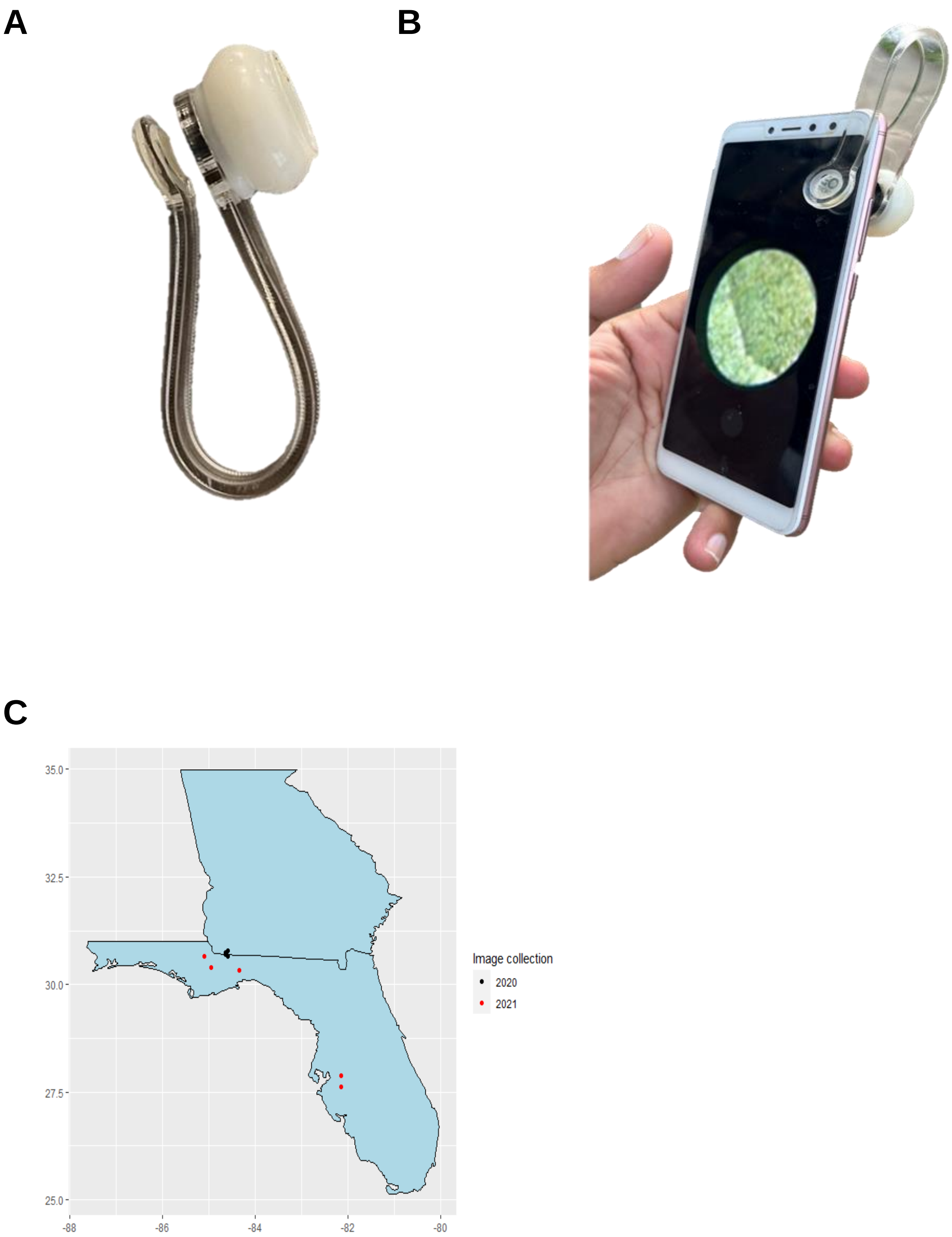

Supplementary Figure 1. Microscopic image collection A. Lens used to caputure microscopic magnifed images B. Lens attached to mobile C. Map of Florida and Georgia showing locations of image collection during year 2020 and 2021.
