# Supplementary material for: Non-coding deep learning models for tomato biotic and abiotic stress classification using microscopic images
Source: Front Plant Sci. 2023 Jan 8;14:1292643. doi: 10.3389/fpls.2023.1292643 (PMC10800394; doi:10.3389/fpls.2023.1292643)
Supplement: Supplementary file 2 [file Image_2.pdf]

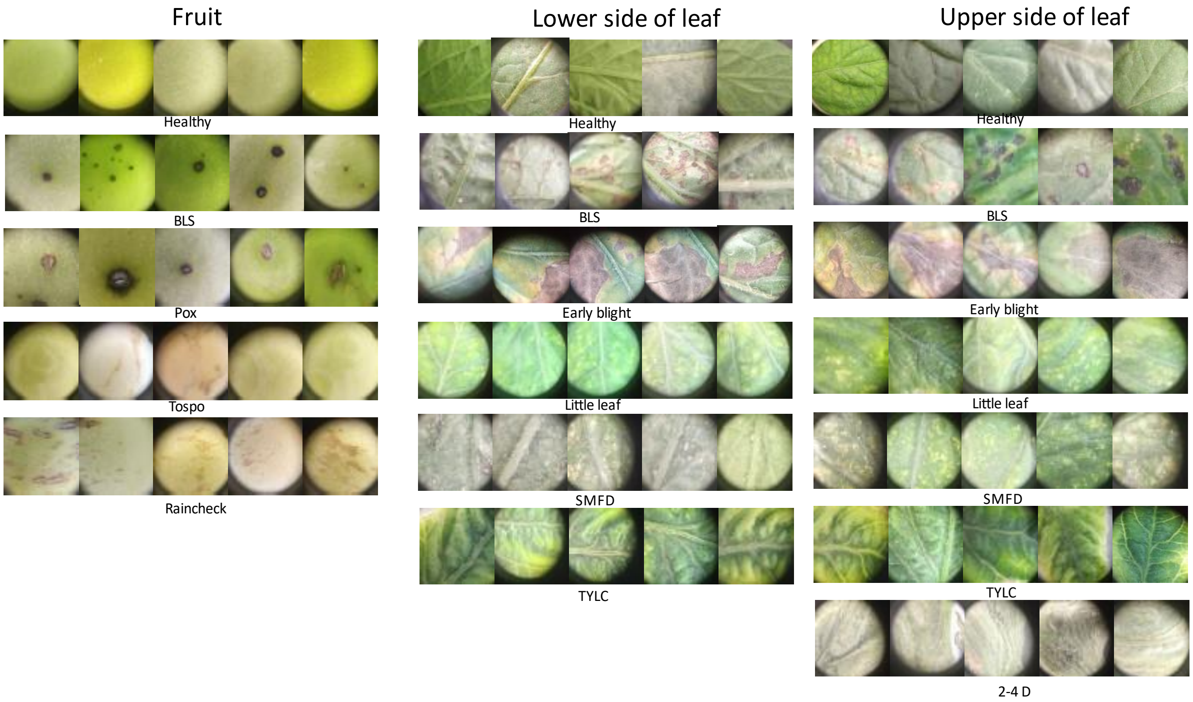

Supplementary Figure 2. Representative microscopic, magnified images for each class taken using 30X lens attached to mobile. SMFD: Spider mite feeding damage; TYLC: Tomato yellow leaf curl; BST: Bacterial spot of tomato; 2-4 D: herbicide 2-4 D spray drift damage symptom; Nutrient: Nutrient deficiency symptom: Tospo: Tomato spotted wilt symptom.
