# Supplementary material for: Non-coding deep learning models for tomato biotic and abiotic stress classification using microscopic images
Source: Front Plant Sci. 2023 Jan 8;14:1292643. doi: 10.3389/fpls.2023.1292643 (PMC10800394; doi:10.3389/fpls.2023.1292643)
Supplement: Supplementary file 3 [file Table_1.docx]

Supplementary Table 1. Diagnostic methods used for validation for disease symptoms

| **Symptom** | **Diagnostic method used for identification** | **Reference** |
| --- | --- | --- |
| Bacterial spot of tomato | Culturing, and PCR | (Strayer et al., 2016) |
| Healthy | No visible symptoms | (Jones et al., 2014) |
| Pox | Expert knowledge | (Jones et al., 2014) |
| Raincheck | Expert knowledge | (Jones et al., 2014) |
| Tomato spotted wilt | Antibody-based test/PCR | (Jones et al., 2014) |
| Early blight | Microscopy, culturing, and PCR | (Jones et al., 2014) |
| Little leaf | Expert knowledge | (Jones et al., 2014) |
| Spider mite feeding damage | Microscopy | (Jones et al., 2014) |
| Tomato yellow leaf curl | PCR | (Jones et al., 2014) |
| Nutrient deficiency | Expert knowledge | (Jones et al., 2014) |
| 2-4-D drift damage | Expert knowledge | (Jones et al., 2014) |
