# Supplementary material for: Non-coding deep learning models for tomato biotic and abiotic stress classification using microscopic images
Source: Front Plant Sci. 2023 Jan 8;14:1292643. doi: 10.3389/fpls.2023.1292643 (PMC10800394; doi:10.3389/fpls.2023.1292643)
Supplement: Supplementary file 4 [file Table_2.docx]

Supplementary Table 2. Machine learning models evaluated for the potential to be used in analysis

| **Model** | **Reason for not using** | **Used in this study** | **Used model URL** |
| --- | --- | --- | --- |
| MakeMl | Don’t provide statistics | Not used |  |
| Lobe | Provide percentage, not much information and data crash with large dataset | Not used |  |
| Baidu EZDL custom Training | Language (Chinese) barrier | Not used |  |
| Datarobot | No image classification | Not used |  |
| DeepCognition | Require significant coding | Not used |  |
| Platform.ai | Difficult to label data | Not used |  |
| ProductAI | Require significant coding | Not used |  |
| Uber Ludwig | Require significant coding | Not used |  |
| IBM Watson visual recognition | Difficult in model evaluation | Not used |  |
| Medicmind | Specifically developed for eye-based images | Not used | https://ai.medicmind.tech/ |
| Teachable machine |  | Used | https://teachablemachine.withgoogle.com/train/image |
| Clarifai |  | Used | https://www.clarifai.com/models/image-recognition-ai |
| Microsoft custom vision |  | Used | https://azure.Microsoft.com/en-us/services/cognitive-services/custom-vision-service/ |
| Apple CreateML |  | Used | https://developer.apple.com/machine-learning/create-ml/ |
| Amazon Recognition custom label |  | Used | https://us-east-1.console.aws.amazon.com/rekognition/custom-labels#/ |
| Google AutoML vision |  | Used | https://console.cloud.google.com/vision/dashboard?projec |
