# Supplementary material for: Non-coding deep learning models for tomato biotic and abiotic stress classification using microscopic images
Source: Front Plant Sci. 2023 Jan 8;14:1292643. doi: 10.3389/fpls.2023.1292643 (PMC10800394; doi:10.3389/fpls.2023.1292643)
Supplement: Supplementary file 5 [file Table_3.docx]

Supplementary Table 3. Comparison of F1 scores of different database pooled images in Non-Coding Deep Learning (NCDL) platforms

| **Database** | **Custom Label** | **Clarifai** | **Teachable Machine** | **AutoML** | **CreateML** | **Custom Vision** |
| --- | --- | --- | --- | --- | --- | --- |
| Fruit | 98.7 | 95.4 | 94.8 | 94.1 | na | 95.5 |
| Lower side of leaf | 100.0 | 94.5 | 94.8 | 97.0 | na | 100.0 |
| Upper side of leaf | 99.2 | 94.1 | 91.2 | 95.5 | na | 99.1 |
| Combined individual classes^a^ | 95.3 | 89.0 | 84.3 | 91.7 | na | 93.0 |
| Leaf image combined^b^ | 99.0 | 95.0 | 91.7 | 96.1 | na | 98.2 |
| Leaf and fruit image combined^c^ | 98.7 | 95.4 | 92.6 | 95.3 | na | 98.9 |
| Average^d^ | 98.5^a^ | 93.9^bcd^ | 91.6^cd^ | 95.0^abc^ | Na | 97.5^ab^ |

^a^: All individual 5 class of fruit, 6 class of lower side of leaf and 8 class of upper side of leaf used. No image class was combined.

^b^: Lower side of leaf class combined with upper side of leaf images class to have single class of images. For example, BST lower side of leaf images combined with upper side of leaf images to have BST(leaf) class. All 6 class to lower side of leaf combined to respective upper side of leaf.

^c^: BST and healthy class upper side of leaf, lower side of leaf and fruit symptom images combined to single class.

^d^: Same letters on average are significantly not different (*P* < 0.05) based on Tukey's multiple comparisons test posthoc analysis. Difference database was treated as replication for statistical analysis.

^b^: Lower side of leaf class combined with upper side of leaf images class to have single class of images. For example, BST lower side of leaf images combined with upper side of leaf images to have BST (leaf) class. All 6 class to lower side of leaf combined to respective upper side of leaf.

^c^: BST and healthy class upper side of leaf, lower side of leaf and fruit symptom images combined to single class.

^d^: Same letters on average are significantly not different (*P* < 0.05) based on Tukey's multiple comparisons test posthoc analysis. Difference database was treated as replication for statistical analysis.
