# Supplementary material for: Non-coding deep learning models for tomato biotic and abiotic stress classification using microscopic images
Source: Front Plant Sci. 2023 Jan 8;14:1292643. doi: 10.3389/fpls.2023.1292643 (PMC10800394; doi:10.3389/fpls.2023.1292643)
Supplement: Supplementary file 6 [file Table_4.docx]

Supplementary Table 4. Pairwise F1 score mean comparison of Non-Coding Deep Learning platform models

| **Tukey's multiple comparisons test** | **Mean Diff.** | **95.00% CI of diff.** | **Below threshold** | **Summary** | **Adjusted P Value** |
| --- | --- | --- | --- | --- | --- |
| Custom Label vs. Clarifai | 4.584 | 0.1384 to 9.030 | Yes | * | 0.0409 |
| Custom Label vs. AutoML | 3.538 | -0.9073 to 7.984 | No | ns | 0.1665 |
| Custom Label vs. Teachable machine | 6.922 | 2.476 to 11.37 | Yes | *** | 0.001 |
| Custom Label vs. Custom Vision | 1.028 | -3.418 to 5.474 | No | ns | 0.9591 |
| Clarifai vs. AutoML | -1.046 | -5.491 to 3.400 | No | ns | 0.9566 |
| Clarifai vs. Teachable machine | 2.338 | -2.108 to 6.783 | No | ns | 0.5449 |
| Clarifai vs. Custom Vision | -3.556 | -8.002 to 0.8898 | No | ns | 0.163 |
| AutoML vs. Teachable machine | 3.383 | -1.063 to 7.829 | No | ns | 0.1999 |
| AutoML vs. Custom Vision | -2.51 | -6.956 to 1.935 | No | ns | 0.4763 |
| Teachable machine vs. Custom Vision | -5.894 | -10.34 to -1.448 | Yes | ** | 0.0054 |
