# Supplementary material for: Non-coding deep learning models for tomato biotic and abiotic stress classification using microscopic images
Source: Front Plant Sci. 2023 Jan 8;14:1292643. doi: 10.3389/fpls.2023.1292643 (PMC10800394; doi:10.3389/fpls.2023.1292643)
Supplement: Supplementary file 7 [file Table_5.docx]

Supplementary Table 5. Accuracy(%) of Non-Coding Deep Learning (NCDL) platform models.

| **Database** | **Custom Label** | **Clarifai** | **Teachable Machine** | **AutoML** | **CreateML** | **Custom Vision** |
| --- | --- | --- | --- | --- | --- | --- |
| Fruit | 99.5 | 98.2 | 97.9 | 97.6 | 88.0 | na |
| Lower side of leaf | 100.0 | 98.2 | 98.3 | 99.0 | 91.0 | na |
| Upper side of leaf | 99.8 | 98.5 | 97.8 | 98.9 | 90.0 | na |
| Combined individual classes^a^ | 99.5 | 98.8 | 98.3 | 99.1 | 82.0 | na |
| Leaf image combined^b^ | 99.9 | 99.2 | 98.7 | 99.4 | 91.0 | na |
| Leaf and fruit image combined^c^ | 99.8 | 99.2 | 98.7 | 99.2 | 82.0 | na |
| Average^d^ | 99.8^a^ | 98.7^a^ | 98.3^a^ | 98.9^a^ | 87.3^b^ | na |

^a^: All individual 5 class of fruit, 6 class of lower side of leaf and 8 class of upper side of leaf used. No image class was combined.
