# Supplementary material for: Non-coding deep learning models for tomato biotic and abiotic stress classification using microscopic images
Source: Front Plant Sci. 2023 Jan 8;14:1292643. doi: 10.3389/fpls.2023.1292643 (PMC10800394; doi:10.3389/fpls.2023.1292643)
Supplement: Supplementary file 8 [file Table_6.docx]

Supplementary Table 6. Pairwise accuracy comparison of Non-Coding Deep Learning platform models

| **Tukey's multiple comparisons test** | **Mean Diff.** | **95.00% CI of diff.** | **Summary** | **Adjusted P Value** |
| --- | --- | --- | --- | --- |
| Custom Label vs. Clarifai | 1.067 | -2.246 to 4.379 | ns | 0.8761 |
| Custom Label vs. Teachable Machine | 1.467 | -1.846 to 4.779 | ns | 0.6934 |
| Custom Label vs. AutoML | 0.8833 | -2.429 to 4.196 | ns | 0.9331 |
| Custom Label vs. CreateML | 12.42 | 9.104 to 15.73 | **** | <0.0001 |
| Clarifai vs. Teachable Machine | 0.4000 | -2.912 to 3.712 | ns | 0.9964 |
| Clarifai vs. AutoML | -0.1833 | -3.496 to 3.129 | ns | 0.9998 |
| Clarifai vs. CreateML | 11.35 | 8.038 to 14.66 | **** | <0.0001 |
| Teachable Machine vs. AutoML | -0.5833 | -3.896 to 2.729 | ns | 0.9848 |
| Teachable Machine vs. CreateML | 10.95 | 7.638 to 14.26 | **** | <0.0001 |
| AutoML vs. CreateML | 11.53 | 8.221 to 14.85 | **** | <0.0001 |
