# Supplementary material for: Non-coding deep learning models for tomato biotic and abiotic stress classification using microscopic images
Source: Front Plant Sci. 2023 Jan 8;14:1292643. doi: 10.3389/fpls.2023.1292643 (PMC10800394; doi:10.3389/fpls.2023.1292643)
Supplement: Supplementary file 9 [file Table_7.docx]

Supplementary Table 7. Pairwise accuracy comparison of Non-Coding Deep Learning platform models

| **Tukey's multiple comparisons test** | **Mean Diff.** | **95.00% CI of diff.** | **Summary** | **Adjusted P Value** |
| --- | --- | --- | --- | --- |
| Fruit vs. Lower side of leaf | -1.592 | -7.518 to 4.334 | ns | 0.9587 |
| Fruit vs. Combined individual classes | 5.035 | -0.8907 to 10.96 | ns | 0.1290 |
| Fruit vs. Leaf image combined | -0.3331 | -6.259 to 5.593 | ns | >0.9999 |
| Fruit vs. Leaf and fruit image combined | -0.5152 | -6.441 to 5.411 | ns | 0.9998 |
| Fruit vs. Upper side of leaf | -0.1550 | -6.081 to 5.771 | ns | >0.9999 |
| Lower side of leaf vs. Combined individual classes | 6.627 | 0.7013 to 12.55 | * | 0.0222 |
| Lower side of leaf vs. Leaf image combined | 1.259 | -4.667 to 7.185 | ns | 0.9850 |
| Lower side of leaf vs. Leaf and fruit image combined | 1.077 | -4.849 to 7.003 | ns | 0.9926 |
| Lower side of leaf vs. Upper side of leaf | 1.437 | -4.489 to 7.363 | ns | 0.9733 |
| Combined individual classes vs. Leaf image combined | -5.368 | -11.29 to 0.5575 | ns | 0.0917 |
| Combined individual classes vs. Leaf and fruit image combined | -5.551 | -11.48 to 0.3755 | ns | 0.0756 |
| Combined individual classes vs. Upper side of leaf | -5.190 | -11.12 to 0.7356 | ns | 0.1103 |
| Leaf image combined vs. Leaf and fruit image combined | -0.1821 | -6.108 to 5.744 | ns | >0.9999 |
| Leaf image combined vs. Upper side of leaf | 0.1781 | -5.748 to 6.104 | ns | >0.9999 |
| Leaf and fruit image combined vs. Upper side of leaf | 0.3602 | -5.566 to 6.286 | ns | >0.9999 |
