# Supplementary material for: Non-coding deep learning models for tomato biotic and abiotic stress classification using microscopic images
Source: Front Plant Sci. 2023 Jan 8;14:1292643. doi: 10.3389/fpls.2023.1292643 (PMC10800394; doi:10.3389/fpls.2023.1292643)
Supplement: Supplementary file 10 [file Table_8.docx]

Supplementary Table 8. Outputs from Non-Coding Deep Learning (NCDL) platforms for leaf image combined dataset^a^.

| **Database** | **Class^b^** | **TP** | **FP** | **FN** | **TN** | **Precision (%)** | **NPV (%)** | **Recall(%)** | **Specificity(%)** | **Accuracy(%)** | **F1 score** |
| --- | --- | --- | --- | --- | --- | --- | --- | --- | --- | --- | --- |
| **Custom Label** | | | | | | | | | | | |
|  | 2-4 D versus other | 48 | 0 | 0 | 740 | 100.0 | 100.0 | 100.0 | 100.0 | 100.0 | 100.0 |
|  | BST (leaf) versus other | 136 | 0 | 1 | 651 | 100.0 | 99.8 | 99.3 | 100.0 | 99.9 | 99.6 |
|  | BST (fruit) versus other | 105 | 1 | 4 | 678 | 99.1 | 99.4 | 96.3 | 99.9 | 99.4 | 97.7 |
|  | Early Blight (leaf) versus other | 137 | 2 | 0 | 649 | 98.6 | 100.0 | 100.0 | 99.7 | 99.7 | 99.3 |
|  | Healthy (leaf) versus other | 63 | 0 | 1 | 724 | 100.0 | 99.9 | 98.4 | 100.0 | 99.9 | 99.2 |
|  | Healthy(fruit) versus other | 11 | 0 | 0 | 777 | 100.0 | 100.0 | 100.0 | 100.0 | 100.0 | 100.0 |
|  | Little leaf (leaf) versus other | 35 | 0 | 0 | 753 | 100.0 | 100.0 | 100.0 | 100.0 | 100.0 | 100.0 |
|  | Nutrient (leaf) versus other | 31 | 0 | 0 | 757 | 100.0 | 100.0 | 100.0 | 100.0 | 100.0 | 100.0 |
|  | Pox versus other | 24 | 4 | 1 | 759 | 85.7 | 99.9 | 96.0 | 99.5 | 99.4 | 90.6 |
|  | Raincheck versus other | 47 | 0 | 0 | 741 | 100.0 | 100.0 | 100.0 | 100.0 | 100.0 | 100.0 |
|  | SMFD (leaf) versus other | 35 | 0 | 0 | 753 | 100.0 | 100.0 | 100.0 | 100.0 | 100.0 | 100.0 |
|  | Tospo versus other | 33 | 0 | 0 | 755 | 100.0 | 100.0 | 100.0 | 100.0 | 100.0 | 100.0 |
|  | TYLC (leaf) versus other | 76 | 1 | 0 | 711 | 98.7 | 100.0 | 100.0 | 99.9 | 99.9 | 99.3 |
|  | Pooled | 781 | 8 | 7 | 9,448 | 99.0 | 99.9 | 99.1 | 99.9 | 99.9 | 99.0 |
| **Clarifai** | | | | | | | | | | | |
|  | 2-4 D versus other | 41 | 0 | 2 | 716 | 100.0 | 99.7 | 95.3 | 100.0 | 99.7 | 97.6 |
|  | BST (leaf) versus other | 133 | 8 | 1 | 617 | 94.3 | 99.8 | 99.3 | 98.7 | 98.8 | 96.7 |
|  | BST (fruit) versus other | 99 | 8 | 8 | 644 | 92.5 | 98.8 | 92.5 | 98.8 | 97.9 | 92.5 |
|  | Early Blight (leaf) versus other | 122 | 3 | 7 | 627 | 97.6 | 98.9 | 94.6 | 99.5 | 98.7 | 96.1 |
|  | Healthy (leaf) versus other | 59 | 0 | 2 | 698 | 100.0 | 99.7 | 96.7 | 100.0 | 99.7 | 98.3 |
|  | Healthy (fruit) versus other | 12 | 0 | 0 | 747 | 100.0 | 100.0 | 100.0 | 100.0 | 100.0 | 100.0 |
|  | Little leaf (leaf) versus other | 28 | 2 | 4 | 725 | 93.3 | 99.5 | 87.5 | 99.7 | 99.2 | 90.3 |
|  | Nutrient (leaf) versus other | 28 | 0 | 1 | 730 | 100.0 | 99.9 | 96.6 | 100.0 | 99.9 | 98.2 |
|  | Pox versus other | 18 | 8 | 7 | 726 | 69.2 | 99.0 | 72.0 | 98.9 | 98.0 | 70.6 |
|  | Raincheck versus other | 46 | 2 | 2 | 709 | 95.8 | 99.7 | 95.8 | 99.7 | 99.5 | 95.8 |
|  | SMFD (leaf) versus other | 32 | 3 | 1 | 723 | 91.4 | 99.9 | 97.0 | 99.6 | 99.5 | 94.1 |
|  | Tospo versus other | 31 | 1 | 2 | 725 | 96.9 | 99.7 | 93.9 | 99.9 | 99.6 | 95.4 |
|  | TYLC (leaf) versus other | 72 | 3 | 1 | 683 | 96.0 | 99.9 | 98.6 | 99.6 | 99.5 | 97.3 |
|  | Pooled | 721 | 38 | 38 | 9,070 | 95.0 | 99.6 | 95.0 | 99.6 | 99.2 | 95.0 |
| **Teachable machine** | | | | | | | | | | | |
|  | 2-4 D versus other | 34 | 6 | 2 | 554 | 85.0 | 99.6 | 94.4 | 98.9 | 98.7 | 89.5 |
|  | BST (leaf) versus other | 92 | 5 | 11 | 488 | 94.8 | 97.8 | 89.3 | 99.0 | 97.3 | 92.0 |
|  | BST (fruit) versus other | 75 | 4 | 7 | 510 | 94.9 | 98.6 | 91.5 | 99.2 | 98.2 | 93.2 |
|  | Early Blight (leaf) versus other | 97 | 9 | 6 | 484 | 91.5 | 98.8 | 94.2 | 98.2 | 97.5 | 92.8 |
|  | Healthy (leaf) versus other | 43 | 1 | 5 | 547 | 97.7 | 99.1 | 89.6 | 99.8 | 99.0 | 93.5 |
|  | Healthy (fruit) versus other | 8 | 0 | 1 | 587 | 100.0 | 99.8 | 88.9 | 100.0 | 99.8 | 94.1 |
|  | Little leaf (leaf) versus other | 22 | 3 | 4 | 567 | 88.0 | 99.3 | 84.6 | 99.5 | 98.8 | 86.3 |
|  | Nutrient (leaf) versus other | 21 | 2 | 2 | 571 | 91.3 | 99.7 | 91.3 | 99.7 | 99.3 | 91.3 |
|  | Pox versus other | 15 | 4 | 4 | 573 | 78.9 | 99.3 | 78.9 | 99.3 | 98.7 | 78.9 |
|  | Raincheck versus other | 35 | 5 | 1 | 555 | 87.5 | 99.8 | 97.2 | 99.1 | 99.0 | 92.1 |
|  | SMFD (leaf) versus other | 26 | 5 | 0 | 565 | 83.9 | 100.0 | 100.0 | 99.1 | 99.2 | 91.2 |
|  | Tospo versus other | 23 | 0 | 2 | 571 | 100.0 | 99.7 | 92.0 | 100.0 | 99.7 | 95.8 |
|  | TYLC (leaf) versus other | 53 | 5 | 4 | 534 | 91.4 | 99.3 | 93.0 | 99.1 | 98.5 | 92.2 |
|  | Pooled | 544 | 49 | 49 | 7,106 | 91.7 | 99.3 | 91.7 | 99.3 | 98.7 | 91.7 |
| **AutoML** | | | | | | | | | | | |
|  | 2-4 D versus other | 21 | 2 | 3 | 382 | 91.3 | 99.2 | 87.5 | 99.5 | 98.8 | 89.4 |
|  | Early Blight (leaf) versus other | 69 | 1 | 0 | 338 | 98.6 | 100.0 | 100.0 | 99.7 | 99.8 | 99.3 |
|  | BST (fruit) versus other | 54 | 5 | 1 | 348 | 91.5 | 99.7 | 98.2 | 98.6 | 98.5 | 94.7 |
|  | Healthy(fruit) versus other | 7 | 0 | 1 | 400 | 100.0 | 99.8 | 87.5 | 100.0 | 99.8 | 93.3 |
|  | BST (leaf) versus other | 68 | 0 | 2 | 338 | 100.0 | 99.4 | 97.1 | 100.0 | 99.5 | 98.6 |
|  | Healthy (leaf) versus other | 32 | 2 | 2 | 372 | 94.1 | 99.5 | 94.1 | 99.5 | 99.0 | 94.1 |
|  | Little leaf (leaf) versus other | 18 | 1 | 1 | 388 | 94.7 | 99.7 | 94.7 | 99.7 | 99.5 | 94.7 |
|  | Nutrient (leaf) versus other | 16 | 0 | 0 | 392 | 100.0 | 100.0 | 100.0 | 100.0 | 100.0 | 100.0 |
|  | Pox versus other | 8 | 0 | 5 | 395 | 100.0 | 98.8 | 61.5 | 100.0 | 98.8 | 76.2 |
|  | Raincheck versus other | 24 | 0 | 0 | 384 | 100.0 | 100.0 | 100.0 | 100.0 | 100.0 | 100.0 |
|  | SMFD (leaf) versus other | 18 | 2 | 0 | 388 | 90.0 | 100.0 | 100.0 | 99.5 | 99.5 | 94.7 |
|  | Tospo versus other | 18 | 2 | 0 | 388 | 90.0 | 100.0 | 100.0 | 99.5 | 99.5 | 94.7 |
|  | TYLC (leaf) versus other | 39 | 1 | 1 | 367 | 97.5 | 99.7 | 97.5 | 99.7 | 99.5 | 97.5 |
|  | Pooled | 392 | 16 | 16 | 4880 | 96.1 | 99.7 | 96.1 | 99.7 | 99.4 | 96.1 |
| **CreateML** | | | | | | | | | | | |
|  | 2-4 D versus other | - | - | - | - | 93.0 | - | 85.0 | - | - | 88.8 |
|  | Early Blight (leaf) versus other | - | - | - | - | 90.0 | - | 96.0 | - | - | 92.9 |
|  | BST (fruit) versus other | - | - | - | - | 93.0 | - | 91.0 | - | - | 92.0 |
|  | Healthy(fruit) versus other | - | - | - | - | 95.0 | - | 95.0 | - | - | 95.0 |
|  | BST (leaf) versus other | - | - | - | - | 81.0 | - | 86.0 | - | - | 83.4 |
|  | Healthy (leaf) versus other | - | - | - | - | 92.0 | - | 100.0 | - | - | 95.8 |
|  | Little leaf (leaf) versus other | - | - | - | - | 90.0 | - | 80.0 | - | - | 84.7 |
|  | Nutrient (leaf) versus other | - | - | - | - | 90.0 | - | 97.0 | - | - | 93.4 |
|  | Pox versus other | - | - | - | - | 71.0 | - | 68.0 | - | - | 69.5 |
|  | Raincheck versus other | - | - | - | - | 98.0 | - | 96.0 | - | - | 97.0 |
|  | SMFD (leaf) versus other | - | - | - | - | 94.0 | - | 89.0 | - | - | 91.4 |
|  | Tospo versus other | - | - | - | - | 92.0 | - | 100.0 | - | - | 95.8 |
|  | TYLC (leaf) versus other | - | - | - | - | 93.0 | - | 91.0 | - | - | 92.0 |
|  | Pooled | - | - | - | - | na |  | na | - | 91.0 | - |
| **Custom Vision** | | | | | | | | | | | |
|  | 2-4 D versus other | - | - | - | - | 98.0 | - | 100.0 | - | - | 99.0 |
|  | Early Blight (leaf) versus other | - | - | - | - | 100.0 | - | 100.0 | - | - | 100.0 |
|  | BST (fruit) versus other | - | - | - | - | 94.5 | - | 95.4 | - | - | 94.9 |
|  | Healthy(fruit) versus other | - | - | - | - | 100.0 | - | 100.0 | - | - | 100.0 |
|  | BST (leaf) versus other | - | - | - | - | 100.0 | - | 98.4 | - | - | 99.2 |
|  | Healthy (leaf) versus other | - | - | - | - | 100.0 | - | 90.9 | - | - | 95.2 |
|  | Little leaf (leaf) versus other | - | - | - | - | 100.0 | - | 97.1 | - | - | 98.5 |
|  | Nutrient (leaf) versus other | - | - | - | - | 100.0 | - | 100.0 | - | - | 100.0 |
|  | Pox versus other | - | - | - | - | 79.2 | - | 79.2 | - | - | 79.2 |
|  | Raincheck versus other | - | - | - | - | 100.0 | - | 100.0 | - | - | 100.0 |
|  | SMFD (leaf) versus other | - | - | - | - | 100.0 | - | 100.0 | - | - | 100.0 |
|  | Tospo versus other | - | - | - | - | 100.0 | - | 100.0 | - | - | 100.0 |
|  | TYLC (leaf) versus other | - | - | - | - | 98.7 | - | 100.0 | - | - | 99.3 |
|  | Pooled | - | - | - | - | 98.2 | - | 98.2 | - |  | 98.4 |

^a^:TP: True Positives; FP: False Positives; FN: False Negatives and TN: True Negatives NPV: Negative Predictive Value

^b^: SMFD: Spider mite feeding damage; TYLC: Tomato yellow leaf curl; BST: Bacterial spot of tomato; 2-4 D: herbicide 2-4 D spray drift damage symptom; Nutrient: Nutrient deficiency symptom: Tospo: Tomato spotted wilt symptom.
