# Supplementary material for: Non-coding deep learning models for tomato biotic and abiotic stress classification using microscopic images
Source: Front Plant Sci. 2023 Jan 8;14:1292643. doi: 10.3389/fpls.2023.1292643 (PMC10800394; doi:10.3389/fpls.2023.1292643)
Supplement: Supplementary file 11 [file Table_9.docx]

Supplementary Table 9. Confusion matrix with Combined individual classes using Clarifai model.

|  | Predicted class | | | | | | | | | | | | | | | | | | | |
| --- | --- | --- | --- | --- | --- | --- | --- | --- | --- | --- | --- | --- | --- | --- | --- | --- | --- | --- | --- | --- |
|  | Class^a^ | SMFD (Lower side) | SMFD (Upper side) | Little leaf (Upper side) | Little leaf (Lower side) | TYLC (Lower side) | TYLC (Upper side) | BST (Lower side) | BST (Upper side) | Early Blight (Lower side) | Early Blight (Upper side) | Healthy (Upper side) | Healthy (Lower side) | 2-4 D | BST (Fruit) | Pox | Raincheck | Healthy(Fruit) | Nutrient (Upper side) | Tospo |
| Actual class | SMFD (lower side) | 17 | 0 | 1 | 0 | 0 | 0 | 0 | 0 | 0 | 0 | 0 | 0 | 0 | 0 | 0 | 0 | 0 | 0 | 0 |
|  | SMFD (Upper side) | 0 | 10 | 0 | 0 | 0 | 0 | 0 | 0 | 0 | 0 | 0 | 0 | 0 | 0 | 0 | 0 | 0 | 0 | 0 |
|  | Little leaf (Upper side) | 1 | 0 | 15 | 2 | 0 | 1 | 0 | 0 | 0 | 0 | 0 | 0 | 1 | 0 | 0 | 0 | 0 | 0 | 0 |
|  | Little leaf (Lower side) | 0 | 0 | 1 | 4 | 0 | 1 | 0 | 0 | 0 | 0 | 0 | 0 | 0 | 0 | 0 | 0 | 0 | 0 | 0 |
|  | TYLC (Lower side) | 0 | 0 | 0 | 0 | 1 | 2 | 0 | 0 | 0 | 0 | 0 | 0 | 0 | 0 | 0 | 0 | 0 | 0 | 0 |
|  | TYLC (Upper side) | 0 | 0 | 0 | 0 | 4 | 59 | 0 | 0 | 0 | 0 | 1 | 0 | 0 | 0 | 0 | 0 | 0 | 0 | 0 |
|  | BST (Lower side) | 0 | 0 | 0 | 0 | 0 | 0 | 24 | 5 | 1 | 1 | 0 | 0 | 0 | 0 | 0 | 0 | 0 | 0 | 0 |
|  | BST (Upper side) | 0 | 0 | 0 | 0 | 0 | 0 | 16 | 73 | 0 | 2 | 0 | 0 | 0 | 0 | 0 | 0 | 0 | 0 | 0 |
|  | Early Blight (Lower side) | 0 | 0 | 0 | 0 | 0 | 0 | 0 | 0 | 34 | 8 | 0 | 0 | 0 | 0 | 0 | 0 | 0 | 0 | 0 |
|  | Early Blight (Upper side) | 0 | 0 | 0 | 0 | 0 | 0 | 0 | 0 | 5 | 72 | 0 | 0 | 0 | 0 | 0 | 0 | 0 | 0 | 0 |
|  | Healthy (Upper side) | 0 | 0 | 0 | 0 | 0 | 0 | 0 | 0 | 0 | 0 | 44 | 2 | 0 | 0 | 0 | 0 | 0 | 0 | 0 |
|  | Healthy (Lower side) | 0 | 0 | 0 | 0 | 0 | 0 | 0 | 0 | 0 | 0 | 1 | 9 | 0 | 0 | 0 | 0 | 0 | 0 | 0 |
|  | 2-4 D | 0 | 0 | 0 | 0 | 0 | 0 | 0 | 0 | 0 | 1 | 1 | 0 | 42 | 0 | 0 | 0 | 0 | 0 | 0 |
|  | BST (Fruit) | 0 | 0 | 0 | 0 | 0 | 0 | 0 | 0 | 0 | 0 | 0 | 0 | 0 | 96 | 6 | 0 | 0 | 0 | 0 |
|  | Pox | 0 | 0 | 0 | 0 | 0 | 0 | 0 | 0 | 0 | 0 | 0 | 0 | 0 | 9 | 15 | 0 | 0 | 0 | 0 |
|  | Raincheck | 0 | 0 | 0 | 0 | 0 | 0 | 0 | 0 | 0 | 0 | 0 | 0 | 0 | 2 | 0 | 48 | 0 | 0 | 0 |
|  | Healthy (Fruit) | 0 | 0 | 0 | 0 | 0 | 0 | 0 | 0 | 0 | 0 | 0 | 0 | 0 | 1 | 0 | 1 | 11 | 0 | 0 |
|  | Nutrient (Upper side) | 0 | 0 | 0 | 0 | 0 | 0 | 0 | 0 | 0 | 0 | 0 | 0 | 0 | 0 | 0 | 0 | 0 | 28 | 0 |
|  | Tospo | 0 | 0 | 0 | 0 | 0 | 0 | 0 | 0 | 0 | 0 | 0 | 0 | 0 | 2 | 0 | 0 | 0 | 0 | 35 |

^a^: SMFD: Spider mite feeding damage; TYLC: Tomato yellow leaf curl; BST: Bacterial spot of tomato; 2-4 D: herbicide 2-4 D spray drift damage symptom; Nutrient: Nutrient deficiency symptom: Tospo: Tomato spotted wilt symptom
