# Supplementary material for: Non-coding deep learning models for tomato biotic and abiotic stress classification using microscopic images
Source: Front Plant Sci. 2023 Jan 8;14:1292643. doi: 10.3389/fpls.2023.1292643 (PMC10800394; doi:10.3389/fpls.2023.1292643)
Supplement: Supplementary file 12 [file Table_10.docx]

Supplementary Table 10. Testing classification matrices of different Non-Coding Deep Learning (NCDL) platform models using leaf image combined dataset model^a^

| **Database** | **Class^b^** | **TP** | **FP** | **FN** | **TN** | **Precision (%)** | **NPV (%)** | **Recall(%)** | **Specificity(%)** | **Accuracy(%)** | **F1 score** |
| --- | --- | --- | --- | --- | --- | --- | --- | --- | --- | --- | --- |
| **Teachable machine** | | | | | | | | | | | |
|  | BST versus other | 30 | 4 | 3 | 163 | 88.2 | 98.2 | 90.9 | 97.6 | 96.5 | 89.6 |
|  | Early blight versus other | 29 | 4 | 3 | 164 | 87.9 | 98.2 | 90.6 | 97.6 | 96.5 | 89.2 |
|  | Healthy versus other | 25 | 8 | 24 | 143 | 75.8 | 85.6 | 51.0 | 94.7 | 84.0 | 61.0 |
|  | TYLC versus other | 20 | 13 | 16 | 151 | 60.6 | 90.4 | 55.6 | 92.1 | 85.5 | 58.0 |
|  | Raincheck versus other | 25 | 8 | 0 | 167 | 75.8 | 100.0 | 100.0 | 95.4 | 96.0 | 86.2 |
|  | SMFD versus other | 26 | 7 | 4 | 163 | 78.8 | 97.6 | 86.7 | 95.9 | 94.5 | 82.5 |
|  | Little leaf versus other | 22 | 11 | 5 | 162 | 66.7 | 97.0 | 81.5 | 93.6 | 92.0 | 73.3 |
|  | Pooled | 177 | 55 | 55 | 1,113 | 76.3 | 95.3 | 76.3 | 95.3 | 92.1 | 76.3 |
| **AutoML** | | | | | | | | | | | |
|  | BST versus other | 32 | 2 | 2 | 164 | 94.1 | 98.8 | 94.1 | 98.8 | 98.0 | 94.1 |
|  | Early blight versus other | 30 | 3 | 1 | 166 | 90.9 | 99.4 | 96.8 | 98.2 | 98.0 | 93.8 |
|  | Healthy versus other | 29 | 3 | 9 | 159 | 90.6 | 94.6 | 76.3 | 98.1 | 94.0 | 82.9 |
|  | TYLC versus other | 24 | 9 | 7 | 160 | 72.7 | 95.8 | 77.4 | 94.7 | 92.0 | 75.0 |
|  | Raincheck versus other | 32 | 1 | 1 | 166 | 97.0 | 99.4 | 97.0 | 99.4 | 99.0 | 97.0 |
|  | SMFD versus other | 28 | 5 | 2 | 165 | 84.8 | 98.8 | 93.3 | 97.1 | 96.5 | 88.9 |
|  | Little leaf versus other | 25 | 8 | 9 | 158 | 75.8 | 94.6 | 73.5 | 95.2 | 91.5 | 74.6 |
|  | Pooled | 200 | 31 | 31 | 1,138 | 86.6 | 97.3 | 86.6 | 97.3 | 95.6 | 86.6 |
| **Custom Vision** | | | | | | | | | | | |
|  | BST versus other | 31 | 3 | 2 | 164 | 91.2 | 98.8 | 93.9 | 98.2 | 97.5 | 92.5 |
|  | Early blight versus other | 31 | 2 | 3 | 164 | 93.9 | 98.2 | 91.2 | 98.8 | 97.5 | 92.5 |
|  | Healthy versus other | 28 | 3 | 11 | 158 | 90.3 | 93.5 | 71.8 | 98.1 | 93.0 | 80.0 |
|  | TYLC versus other | 27 | 6 | 6 | 161 | 81.8 | 96.4 | 81.8 | 96.4 | 94.0 | 81.8 |
|  | Raincheck versus other | 32 | 1 | 0 | 167 | 97.0 | 100.0 | 100.0 | 99.4 | 99.5 | 98.5 |
|  | SMFD versus other | 28 | 5 | 1 | 166 | 84.8 | 99.4 | 96.6 | 97.1 | 97.0 | 90.3 |
|  | Little leaf versus other | 26 | 7 | 4 | 163 | 78.8 | 97.6 | 86.7 | 95.9 | 94.5 | 82.5 |
|  | Pooled | 203 | 27 | 27 | 1,143 | 88.3 | 97.7 | 88.3 | 97.7 | 96.1 | 88.3 |

^a^:TP: True Positives; FP: False Positives; FN: False Negatives and TN: True Negatives NPV: Negative Predictive Value

^b^: SMFD: Spider mite feeding damage; TYLC: Tomato yellow leaf curl; BST: Bacterial spot of tomato; 2-4 D: herbicide 2-4 D spray drift damage symptom; Nutrient: Nutrient deficiency symptom: Tospo: Tomato spotted wilt symptom.
